# Hereditary Spiny Keratoderma in a Pediatric Patient: A Case Report and Literature Review

**DOI:** 10.7759/cureus.74789

**Published:** 2024-11-29

**Authors:** Joyce Andrea Erize Herrera, Lexli Danae Pacheco Santiago, Diego Olin Pérez Rojas, Esther Guadalupe Guevara Sanginés

**Affiliations:** 1 Dermatology, Institute for Social Security and Services for State Workers Regional Hospital "Lic. Adolfo Lopez Mateos", Mexico City, MEX; 2 Internal Medicine, Institute for Social Security and Services for State Workers Regional Hospital "Lic. Adolfo Lopez Mateos", Mexico City, MEX

**Keywords:** dermatoscopy, hyperkeratosis, keratoderma spiny, keratotic lesions, neoformations

## Abstract

A 14-year-old male with disseminated superficial porokeratosis and a family history of the same lesions on his maternal side presented with spiny keratoderma. Spiny keratoderma is a dermatosis characterized by multiple punctate keratotic neoformations on the palms and soles. It is considered a rare disease, with fewer than 84 cases reported in the world medical literature to date.

## Introduction

Spiny keratoderma, also known as porokeratosis palmaris et plantaris, is a rare entity characterized by the presence of multiple filiform hyperkeratotic neoformations on the palms and soles [1]. A meticulous physical examination of these lesions and adequate lighting is necessary for better visualization. This condition, classified as part of the palmoplantar keratodermas, can be familial or acquired and may be associated with underlying diseases such as oncological pathology, hereditary disorders, sporadic conditions, or post-inflammatory diseases. It manifests as the characteristic coronoid lamellae, the histopathological finding of focal columns of ortho or parakeratotic hyperkeratosis, associated with hypogranulosis and a depressed epidermis [2].

## Case presentation

**Figure 1:**
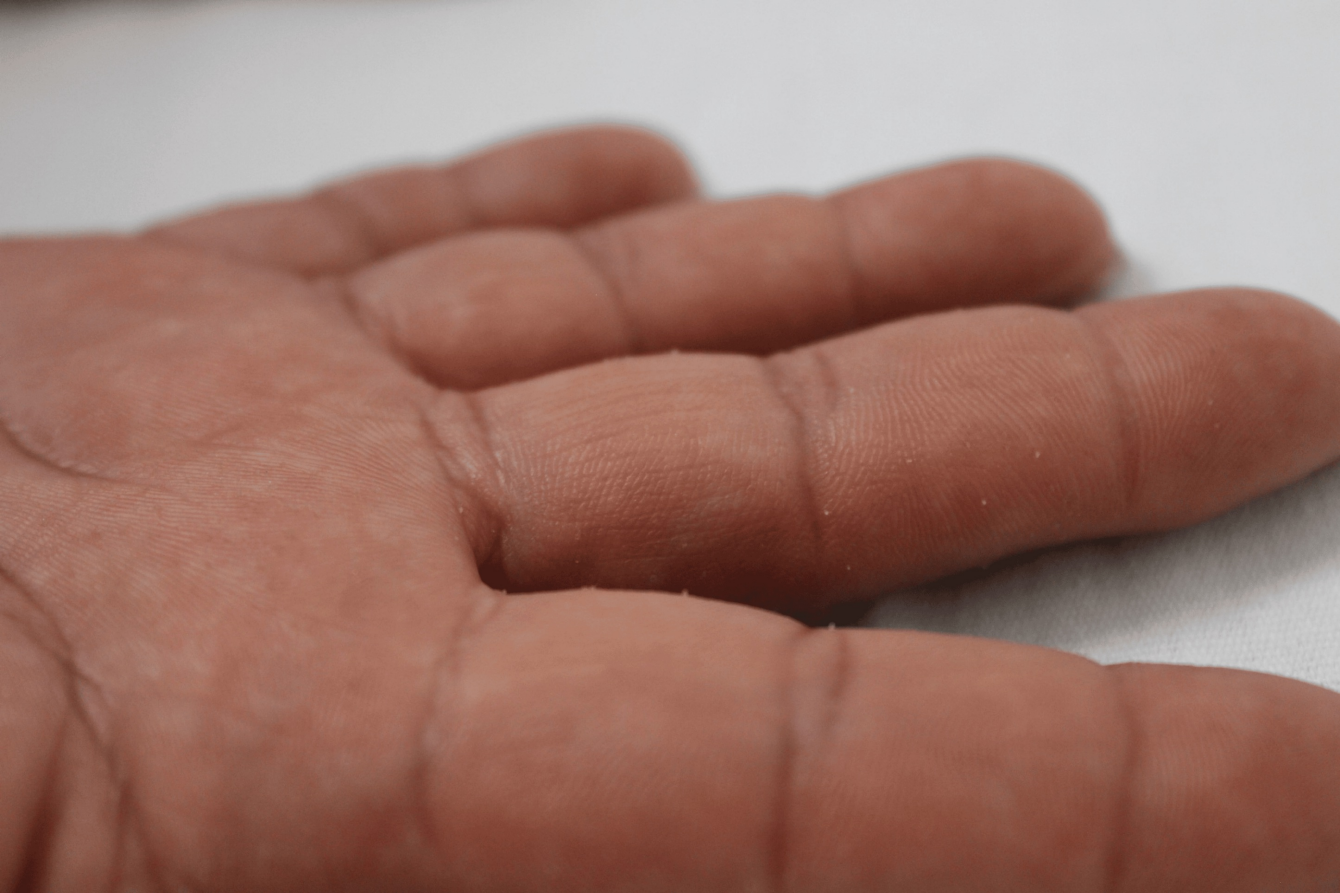
Multiple millimetric filiform keratotic neoformations on palms

A 14-year-old male adolescent presented to our hospital with persistent skin lesions. The patient had a history of renovascular systemic arterial hypertension. In addition, his mother and six maternal relatives had similar skin lesions. He reported a one-year evolution of gradual, mildly painful, disseminated, bilateral, and symmetrical dermatitis affecting both upper extremities on the palms. This was characterized by multiple neoformations of millimetric oval keratotic lesions, some of which had converged to form larger, skin-colored plaques with well-defined edges, rough to palpation (Figure 1).

**Figure 2:**
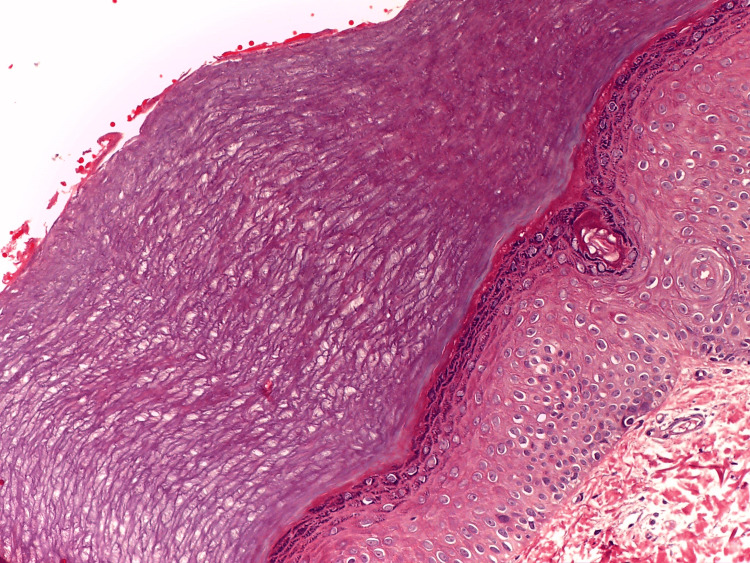
Orthokeratotic hyperkeratosis associated with focal hypogranulosis

Dermatoscopy revealed multiple filiform, non-melanocytic lesions with several small, white, keratotic, exophytic cylindrical projections resembling spines. Blood tests were performed, and no abnormalities were detected. An excisional biopsy of the thenar region was performed with a 4-mm punch, with data compatible with keratoderma spinulosa (predominantly focal hyperkeratosis associated with focal hypogranulosis) (Figure 2).

The treatment started with 40% urea cream every 12 hours for six months, with 90% improvement in the lesions at two months follow-up.

## Discussion

This dermatosis is characterized by multiple punctate keratotic neoformations on the palms and soles [1]. This disease, which has been considered part of the palmoplantar keratodermas, has been classified within the digitate keratosis spectrum and has received multiple names throughout its history, such as music box dermatosis, punctate porokeratotic keratoderma, and plantar filiform hyperkeratosis. The first case was originally described in 1971 by Brown. To date, around 50 cases have been reported. One-third of patients with a history of malignant neoplasia have been reported [3,4].

Its etiology is not well elucidated; however, it is believed that it may be secondary to some alteration in keratinization [5], with four possible theories: (1) Ectopic hair formation based on a hair-specific antibody (pancytokeratin or AE13); (2) increased proliferation secondary to overexpression of keratins 6 and 16 (markers of hyperproliferative cells) attributable to chronic trauma; (3) increase in the proliferation of keratinocytes due to overexpression of p63, a transcription factor that regulates their proliferation and differentiation; and (4) induction of epidermal hyperplasia due to an alteration of cholesterol synthesis caused by coenzyme A reductase inhibitors [6].

Regarding its epidemiology, it is considered a rare disease. Most of the reports come from the United States, with a greater impact on men with a 1:3 ratio [4,7]. This can be found as hereditary (30%) or acquired (70%) pathology. Hereditary cases are usually associated with an autosomal dominant pattern, while acquired ones can be found in conjunction with malignant neoplasms (28.6%), mainly skin cancers, clear cell renal carcinoma, lung adenocarcinoma, and colon adenocarcinoma. Other systemic diseases include asthma, hypertension, type 2 diabetes, polycystic kidney disease, and hyperlipoproteinemia, due to exposure to asbestos or arsenic [4]. The course is not always parallel to the neoplasia, and some cases precede the appearance of the neoplasia by up to 30 years [8]. Based on these presentations, it is understandable that two peaks of incidence are found: the first (mostly related to hereditary cases) at 20 years of age and the second at 60 years of age (related to acquired cases).

Diagnostic challenges regarding the clinical picture are classically described topography as it affects both palms and soles, as well as its morphology consisting of countless punctate hyperkeratotic filiform neoformations. Sometimes, mainly in initial lesions, these can be difficult to identify with the naked eye; therefore, dermoscopy can help us by revealing the presence of a non-melanocytic lesion composed of multiple round, yellowish-white hyperkeratotic spicules arising from both the grooves and ridges [4].

Although clinical diagnosis is highly accurate, a histopathological study of the lesion must be performed to confirm this, where the presence of localized hyperkeratosis composed of columns of parakeratotic cells (chimney lesions) with underlying hypo or agranulosis will be observed, generating a lower columnar depression very similar to the cornoid lamella of porokeratosis.

It is crucial to conduct a thorough diagnostic evaluation in patients suspected of having acquired spiny keratoderma to rule out associated diseases. This should include a comprehensive personal and family medical history, along with regular and close periodic follow-ups, as there is evidence of associations with malignant conditions [9,10].

The differential diagnoses will be given by disseminated palmoplantar porokeratosis, arsenical keratosis, multiple filiform warts, keratoderma puntata palmoplantaris, and acrokeratoelastoidosis. To differentiate spiny keratoderma from porokeratosis, the latter presents vacuolated or dyskeratotic cells in the spinous layer. Regarding the differences with arsenical keratoses, the lesions of arsenical keratosis tend to be more spiculated and discreet [11,12].

As it is such a rare disease, there are no established treatment guidelines for it. However, the use of initial topical treatment based on retinoids or emollients containing keratolytics such as salicylic acid, urea, ammonium lactate, or propylene glycol is common. In cases of persistent lesions, systemic retinoids have been used with variable success rates [13].

## Conclusions

In conclusion, the presented case of disseminated superficial porokeratosis highlights the intricate clinical course and therapeutic challenges associated with this rare dermatological disorder. While this is a hereditary case, non-hereditary cases are often associated with systemic diseases, primarily neoplasms. Therefore, the diagnostic approach should prioritize ruling out these conditions.
